# Linkage of strata of forest vegetation with forest soil microbiomes: a review

**DOI:** 10.3389/fmicb.2025.1575691

**Published:** 2025-06-23

**Authors:** Frank S. Gilliam

**Affiliations:** Department of Earth and Environmental Sciences, University of West Florida, Pensacola, FL, United States

**Keywords:** soil microbiome, forest overstory, herbaceous layer, forest soil, linkage

## Abstract

A major dimension of pattern and process in ecological systems is the way in which species interact. In the study of forest communities, the phenomenon of linkage among forest strata (e.g., overstory and herbaceous layer) has been well investigated and arises when forest strata interact in ways that lead to causal connections between them. Whereas trees alter the light regime of forest herb communities, the herb layer can direct survivorship among seedlings of overstory species. Less studied, however, is linkage between forest strata and forest soil microbiomes. This review examines ways in which forest vegetation and soil microbiomes exert reciprocating effects on each other that can lead to linkage, beginning with a brief literature review of several phenomena relevant to how these effects occur. Because of the coincidence of the ubiquity of soil microbes with their almost infinitely small size, their interactions—both above and belowground in nature—with forest vegetation are particularly intimate. Although the most direct link, and certainly one that likely first comes to mind, is through root/microbe interactions, foliar surfaces and internal foliar tissues can support a diverse microbiome. Following the overview of potential mechanisms, examples from two separate forest studies of how linkage was demonstrated will be summarized. In each of these studies, linkage was evident through significant correlations among axis scores generated by canonical correspondence run separately for forest vegetation and soil microbial communities.

## Introduction

The concept of pattern and process has been of central focus in the study of ecological systems since the early days of the field of ecology (Watt, 1947; Hutchinson, 1953), which Levin (1992) identified as a central problem in the study of ecology, uniting subdisciplines ranging from population ecology to ecosystem ecology, and spanning spatial scales from individuals up to landscapes (Turner, 1989; Murrell et al., 2001; Gilliam, 2022). At the community level, such as a forest, a major dimension of pattern and process in ecological systems is the way in which species—often seemingly unrelated—interact. Interactions in this context refer to mutual, synergistic effects that organisms have on each other.

In the study of forest communities, with their often numerous strata (i.e., layers) which are created and maintained by variation in life history and resource requirement, the phenomenon of linkage among forest strata (e.g., overstory and herbaceous layer) has been well investigated (Gilliam and Roberts, 2014). Linkage arises when forest strata interact in ways that lead to causal connections between them (Gilliam and Roberts, 2014). For example, whereas trees alter the light regime of forest herb communities (Neufeld and Young, 2014), the herb layer can, in turn, direct survivorship among seedlings of overstory species (George and Bazzaz, 2014). When this occurs, the strata are said to exhibit linkage.

These mechanisms to explain linkage between vegetation strata have been demonstrated in the literature for hardwood and conifer forests alike (Barbier et al., 2008; Chandy and Gibson, 2009; Chávez and Macdonald, 2010), with several emphasizing the importance of disturbance (Roberts and Gilliam, 2014) in altering overstory/herb layer interactions (Ellum et al., 2010; Belote et al., 2009; Fleming and Baldwin, 2008; Durak, 2012). Less studied, however, is linkage between forest strata and forest soil microbiomes.

Forest ecosystems have been described as comprising a paradox of biodiversity (Gilliam et al., 2023). The woody overstory that is their most apparent component is, by far, the least diverse with respect to numbers of species. By contrast, the herbaceous layer is the plant community of the most diminutive physical stature, but typically represents 80–90% of forest plant diversity (Gilliam, 2007, 2014). Even less apparent, however, is the biotic community of greatest diversity—*the forest microbiome* (Mishra et al., 2020; Uroz et al., 2016), most particularly that of forest soil (Ji et al., 2022). Van der Heijden et al. (2008) estimated that 1 g of soil can support 10^10^–10^11^ bacteria from between 6,000 and 50,000 species.

Because of the coincidence of the ubiquity of soil microbes with their almost infinitely small size, their interactions—both above and belowground in nature—with forest vegetation are particularly intimate. Thus, as will be examined herein, the potential is great for forest vegetation and soil microbial communities to establish and maintain causal linkages. Although the most direct link, and certainly one that likely first comes to mind, is through root/microbe interactions, foliar surfaces and internal foliar tissues also can support a diverse microbiome (Vorholt, 2012).

This review examines ways in which forest vegetation and soil microbiomes can exert reciprocating effects on each other in ways that can lead to linkage, beginning with a brief literature review of several phenomena relevant to how these effects occur. Although not exclusively so, emphasis will be placed on more recent literature on such processes.

After describing methodologies used to assess linkage in forest ecosystems, two published case studies that specifically examined linkage between forest plant communities and soil microbiomes are summarized. Although these two studies represent sharply contrasting forest types (and, indeed, different research questions), both found evidence of vegetation/microbiome linkage. Finally, directions for future research will be discussed.

## Overstory/microbiome interactions

### Phyllosphere microbial communities

Although feedback between the forest overstory and microbial communities is likely more often considered as part of complex belowground dynamics, and indeed that is the ultimate focus of this review, aboveground interactions—particularly involving foliar surfaces and tissues, the *phyllosphere*—are also important and relevant to this general topic (Bashir et al., 2022). Even though they are not as widely studied as their root-based counterparts, the phyllosphere microbiome is essential on a variety of scales, from individual plant health to ecosystem function (Duan et al., 2024). Vorholt (2012) reported that the total global leaf area is approximately twice that of the total land area (i.e., a global leaf area index of 2), which results in leaf epiphytic microbes numbering up to 10^26^ bacterial cells. Although the fungal component of the phyllosphere microbiome has not been estimated (and is indeed less studied than that of bacteria—Köhler et al., 2025), it is expected to be lower than that of bacteria (Lindow and Brandl, 2003). In sharp contrast with the rhizosphere (Han et al., 2022), the phyllosphere microbiome is typically dominated by a few bacterial phyla that exhibit specific adaptations and relationships with the host plants (Vorholt, 2012).

In an extensive review, Vandenkoornhuyse et al. (2015) developed the concept of the plant holobiont, observing that plants are capable of hosting a broad diversity of microbial organisms inside their tissues (the *endosphere*) and on their surfaces, especially foliar epidermis tissue (the *ectosphere*). These microbes contribute to essential functions of the plant, including plant nutrition, as well as resistance to biotic and abiotic stressors. The plant holobiont, then, comprises the plant itself along with the entire plant microbiome, with its effect on plant fitness via growth and survival. Vandenkoornhuyse et al. (2015) further defined the core microbiome by shared predictive functions.

Duan et al. (2024) reviewed several environmental influences on microbial communities of the phyllosphere, particularly those that vary with elevation. Part of their work emphasized comparisons of the core microbiomes of six ecologically important tree species in temperate forests of Europe sampled from two national parks, one in each of Germany and The Netherlands, with a specific focus on European beech (*Fagus sylvatica*) and Norway spruce (*Picea abies*). They found that bacterial communities were determined mostly by host species, although there was great variation within beech and spruce trees. They further discovered that there was a core microbiome characteristic of all species studied, although community composition varied with elevation, tree diameter, and leaf traits, such as chlorophyll and P content. Ultimately, and closely related to the context of linkage between vegetation strata and soil microbiomes, they clearly demonstrated the intimate association between the phyllosphere microbial communities in forests with host trees. Thus, as leaves senesce and are dropped to the forest floor, they provide what is essentially a species-specific inoculum of microbes to the O horizon and the mineral soil.

Another facet of microbiomes more commonly associated with soil dynamics—nitrification—has also been found to occur in the phyllosphere. Nitrification is carried out only by specialized taxa of bacteria and archaea, beginning with the oxidation of ammonium (NH_4_^+^) to nitrite (NO_2_^−^) and followed by further oxidation to nitrate (NO_3_^−^). This is an important component of nitrogen biogeochemistry in many forest ecosystems (Barbour et al., 1999). Recent work by Guerrieri et al. (2019, 2024) has demonstrated not only presence of nitrifier bacteria and archaea as a notable part of phyllosphere microbiomes, but has quantified the amount of nitrification that occurs in tree canopies. On a process level, they found that 80% of NO_3_^−^ reaching the forest floor in throughfall was from net nitrification in the canopy (Guerrieri et al., 2024). They also identified phyllosphere bacterial taxa as particularly enriched in phyla from the Bacteroidetes and Actinobacteria, two groups closely associated with NH_4_^+^ oxidation (Guerrieri et al., 2019; Yue et al., 2024).

In addition to the phyllosphere microbiome involved in the N biogeochemistry of forest ecosystems, the phyllosphere is also host to methanotrophs, especially those of the genera *Methylobacterium*, *Methylomonas*, *Methylosinus*, and *Methylocystis*. *Methylobacterium* is the primary component of the phyllosphere, whereas *Methylomonas*, *Methylosinus*, and *Methylocystis* are also found in the rhizosphere, which further contributes to the linkage of above- and belowground processes (Iguchi et al., 2012).

It is clear that the phyllosphere of forests hosts a broad microbiome, one that, despite its minimal relative biomass, serves several functions essential to ecosystem structure and function. This also represents a conduit leading to functional linkage between the forest overstory and soil microbiomes essentially by serving as a source of inoculum as leaves senesce and drop to the floor on an annual basis. In the case of temperate deciduous forests, this input comprises the entirety of the phyllosphere during autumn leaf fall.

### Rhizosphere/root exudates

In many ways, the rhizosphere is the belowground equivalent, or certainly analog, of the phyllosphere of forest ecosystems (Enea et al., 2025). Among the sharp contrasts between the phyllosphere and rhizosphere is that the fine roots that comprise the bulk of the rhizosphere continually produce exudates, a complex mixture of organic compounds and inorganic ions that microbes use in a variety of ways. It has been known for quite some time that root exudates represent an important component in the biogeochemistry of forest ecosystems. For example, Smith (1976) identified >15 amino acids/amides, four carbohydrates, eight organic acids, and numerous cations and anions in root exudates from ecologically important tree species of northern hardwood forests. He further found a notable degree of variation among species, suggesting that the biochemical character of root exudates was species specific. Especially regarding the organic compounds—which were the most abundant component, particularly organic acids—this last observation has important implications for linkage between forest vegetation and soil microbiomes.

Indeed, using natural abundance isotope analysis of individual microbial groups, Thacker and Quideau (2021) found a significantly higher proportion of fungi and a higher gram-negative to gram-positive bacteria ratio in the rhizosphere compared to bulk soil. Fungi and gram-negative bacteria biomarkers in the rhizosphere were depleted in ^13^C relative to bulk forest floor, suggesting that microbes assimilated more newly-photosynthesized carbon than did bulk forest floor microbes. More relevant to the question of linkage, they also found sharp species-specific differences in these patterns, with aspen trees influencing their rhizospheres more greatly than did spruce trees relative to microbial community composition and functional capacity. Furthermore, basal respiration was significantly higher in aspen versus spruce rhizospheres.

Other recent work has emphasized the connection between soil microbial activity in the rhizosphere and strategies for above-and belowground resource acquisition by plants. Han et al. (2022) investigated mixed evergreen broadleaf forest of subtropical China, focusing on a variety of soil microbial activities in the rhizosphere, along with soil properties. These activities were characterized primarily via determination of several extracellular catalytic enzymes associated with microbes. Further, they compared leaf and root traits of >20 woody species common in these forest stands and found a notable diversity in microbial activity, with species-specific variation associated with plant growth strategies (i.e., fast-versus slow-growing plants). In particular, soil microbes associated with the rhizosphere of fast-growing plant species exhibited higher metabolism than that of slow-growing plant species (Han et al., 2022). Again, the relevance of these patterns to linkage between forest vegetation and soil microbiomes is seen in their species-specific nature.

Guo et al. (2023) investigated the connection between soil microbial diversity and plant community composition in Masson pine (*Pinus massoniana*) forests of southeastern China (Zhejiang Province), distinguishing between bacterial and fungal functional groups. Masson pine is a prominent component of early successional forests throughout much of China (Lu et al., 2011), and they found sharp contrasts in the factors affecting bacteria versus fungi. Bacterial groups responded negatively to soil N and P as they metabolized root exudates including carbohydrates and amino acids, whereas fungal groups—both saprophytic and parasitic—were negatively correlated with woody species composition (Guo et al., 2023).

As it was with the phyllosphere, it is clear that rhizosphere dynamics, especially as related to root exudates, represent a functional mechanism to explain linkage between forest vegetation and soil microbiomes. Of particular importance in this context is the often species-specific nature of the biochemical composition of root exudates and the microbial groups that use them for their energetic and nutritional requirements.

### Mycorrhizal relationships

Much has been written on the mutualistic relationship between certain fungal taxa and the roots of vascular plants known as *mycorrhizae*, with research and reviews focusing on multiple facets of mycorrhizal dynamics, including their significance on the ecosystem scale (Frey, 2019; Pries et al., 2022). Indeed, it is quite common among plant species. Wang and Qiu (2006) estimated that 80 and 92% of terrestrial plant species and families, respectively, are mycorrhizal. They also found that arbuscular mycorrhiza (AM) is the predominant and ancestral mycorrhizal type among terrestrial plants, suggesting that the origin of AM likely coincided with the origin of land plants. Finally, and more relevant to this discussion of linkage, is that the coevolution between plants and fungi of ectomycorrhizal partnerships likely contributed to diversification of both plant hosts and fungal symbionts (d’Entremont and Kivlin, 2023).

Although AM has been shown to exert profound effects on soil bacteria—especially those associated with N biogeochemistry (Zhang et al., 2022; He et al., 2023; McPherson et al., 2024)—and fungi (Fitch et al., 2023; Noguchi and Toju, 2024), of particular importance to this discussion of linkage between forest vegetation and soil microbiomes is the relationship between forest composition and mycorrhizae. In fact, numerous studies have quantified the causal connection between tree species composition/richness and mycorrhizal associations. Zheng et al. (2023) synthesized the results of >50 peer-reviewed studies to examine the influence of tree species mycorrhizal association on microbial-mediated enzyme activity and stoichiometry. They found sharp contrasts between type of association [arbuscular mycorrhiza (AM) versus ectomycorrhiza (ECM)] and tree phylogenetic group (conifer versus hardwood). Ma et al. (2024) assessed tree species richness and biomass as they vary among AM-and ECM-dominated forest stands of temperate and subtropical China. They found that stands dominated by either type of association supported lower tree species richness and biomass than stands with a relatively equal mixture of associations, concluding that mycorrhizal dominance influences tree species richness and the relationship between richness and biomass in forests of China.

d’Entremont and Kivlin (2023) reviewed hypotheses that have been proposed to explain plant host specificity involved in both AM and ECM associations, namely the Passenger, Driver, and Habitat Hypotheses. Whereas the Passenger Hypothesis posits that the fungal symbionts are somewhat passive in the relationship, being affected more by the host plant, the Driver Hypothesis views fungal symbionts as quite active in determining plant species characteristics, including species composition. By contrast, the Habitat Hypothesis considers neither plant nor fungi to be active, rather viewing environmental gradients as controlling covariation of these communities.

A distinct type of mycorrhizae—ericoid mycorrhizae (ErM)—exhibits characteristics particularly relevant to linkage, considering that it involves (1) fungi primarily of the Ascomycota (but also some Basidiomycota) and (2) roots of species of the Ericaceae; regrettably, ErM is also the least researched of mycorrhizal types (Vohník, 2020). This mutualism allows ericaceous species to occupy the typically highly acidic and infertile soils characteristic of the family. Fungal symbionts secrete organic acids that limit microbial activity, especially those involved with N cycling and including nitrifying bacteria (Straker, 1996), thus altering the composition of the herbaceous layer of forests, as well as the dynamics of tree regeneration (Gilliam et al., 2005). Working in boreal forest soils, Timonen et al. (2017) found that ErM mycospheres influence plant-specific bacterial communities, concluding that the occurrence of ericoid plants, by nature of their fungal symbionts, increases overall bacterial diversity. On the other hand, Adeoyo et al. (2019) demonstrated that some ErM fungi exhibit anti-bacterial properties. Thus, mycorrhizae can simultaneously affect the composition of plant species, as well as the communities of soil microbes.

## Herbaceous layer/microbiome interactions

In addition to the overstory, forest herb communities can establish linkage with soil microbiomes. Indeed, virtually all of the phenomena described herein for overstory/microbial interactions—phyllosphere, rhizosphere, and mycorrhizae—are relevant for the herb layer (Helgason et al., 1999; Hewins et al., 2015).

It has been known for some time that some herbaceous species associated with forest herb communities, especially grasses, legumes, and members of the Convolvulaceae (morning glory family), establish a heritable endosymbiosis with fungi known as *fungal endophytes* (Clay, 1990). Furthermore, these fungal endophytes produce secondary metabolites that are bioactive (Quach et al., 2023). Among their many effects, endophytic secondary metabolites have been shown to directly influence soil microbes, especially those associated with soil N processing (Chen et al., 2022).

Recent research has stressed the interactive dynamics between forest herb communities and soil microbiomes. Working in numerous forest plantation sites in subtropical China, Yin et al. (2016) found evidence that the understory herb layer (as characterized by biomass and species richness) exerted strong controls on soil microbial communities, more so even than tree cover and several soil variables, such as organic C and pH. Chen et al. (2021) distinguished between the potential influence of herbs versus shrubs on soil microbial communities, concluding that roots of herbs affected bacteria communities, whereas roots of shrubs affected fungal communities.

Focusing on AM fungal abundance, Zubek et al. (2021) found notable variation in forest habitat (i.e., beech versus riparian forests of Poland) to drive the effects of herbs with contrasting traits on AM. Zubek et al. (2024) extended the scope of that investigation to include the influence of herbs on the spatial heterogeneity of forest soil via effects on fungal and bacterial diversity. They concluded that herbs affected the composition of AM fungi community in the beech forest, whereas they affected endophytes and plant pathogens in the riparian forest. These results were supported by further investigations that used a factorial field design to determine the impact of ecologically important species of forest and riparian herb communities on soil conditions and microbial communities (Stefanowicz et al., 2022, 2023), finding that forest herbs generally had positive effects on soil microbes by supporting microbial performance.

## Case studies—evidence of linkage

Given the evidence presented here for numerous pathways of interchange between forest vegetation and soil microbial communities, it should not be surprising that there is evidence of linkage between the two. This final section synthesizes the findings of two published works to be used as case studies specifically for how linkage can be assessed. Other than their objective to describe forest vegetation/soil microbiome linkage being in common (along with identical analytical approaches to assess linkage), they represent fully independent investigations, asking different ecological questions, using different field and microbial analytical methodologies, and being carried out at sharply contrasting temperate forest sites—one in West Virginia (Gilliam et al., 2014), the other in Florida (Gilliam et al., 2023). These will be summarized and compared with brief descriptions of study sites, field sampling, data analysis, and findings. Readers are encouraged to access the original publications for more detailed information regarding site, sampling, and analyses.

The West Virginia study was on both sides of an upland ridge dominated by mixed hardwood stands, whereas the Florida study sampled within hardwood-versus longleaf pine-dominated stands. Thus, their central question regarding linkage was different, with the West Virginia study asking whether linkage varied with slope aspect and the Florida asking if linkage varied with stand type. Additionally, the West Virginia study included sampling the herbaceous layer, whereas the Florida did not. Finally, although the plot size and forest overstory sampling were identical between the two studies, the methodologies used to characterize the soil microbiome were fundamentally different. The West Virginia study used phospholipid fatty acid (PLFA) analysis to determine microbial functional groups, a method that was once more common but is still used in current research (e.g., Stefanowicz et al., 2023). The Florida study used high-throughput sequencing of DNA extracted from mineral soil to characterize bacterial composition.

### Study sites

The West Virginia study was carried out at the Beech Fork Lake State Wildlife Area, located in Wayne County, West Virginia (38° 18′N, 82° 25′W), with sampling carried out on a ridge with slopes of roughly north/northeast (N) and south/southwest (S) aspects; elevation ranges of the areas sampled were approximately 178 m to 237 m above mean sea level. The Florida study was carried out in forested stands within the Campus Side Trails area of the University of West Florida, Pensacola, Florida (30° 33′8” N, 87° 13′29” W), focusing on two specific stand types—hardwood dominated versus longleaf pine dominated stands—with elevation 2–5 m above mean sea level.

### Field sampling

Both studies sampled within circular 400-m^2^ plots. For the West Virginia study, these were located in a grid of four plots along each of four parallel transects extending from the N-to S-facing aspect of the ridge, yielding eight plots per aspect. For the Florida study, there were 12 plots in each of hardwood-and pine-dominated stands. All living trees ≥2.5 cm diameter at breast height (DBH) within each plot were identified to species and measured for DBH to the nearest 0.1 cm. In addition for the West Virginia study, the herbaceous layer was assessed by identifying all species of vascular plants ≤1.0 m in height within the entire plot. Abundance of each species was visually estimated using a modified Daubenmire cover scale (Barbour et al., 1999).

For both studies, mineral soil was taken to a 5-cm depth from within each plot and placed in two sterile polyethylene Whirl-Pac® bags, one bag for determination of soil microbial community structure (phospholipid fatty acid analysis for the West Virginia study, extractable DNA for the Florida study) and the other for nutrient analyses. Equipment used for sampling was sanitized between sample plots with a 70% ethanol solution.

### Data analyses

To examine plant species, microbial functional groups, and environmental contrast, quantitative data for all groups were analyzed separately along with a variety of soil variables, including moisture, organic matter, and inorganic and mineralizable N, using canonical correspondence analysis (CCA) (Canoco for Windows 4.5). Linkage among plant and microbial communities was assessed by performing CCA on community/soil data for each slope aspect separately. Linkage was determined via correlation among CCA axis scores for overstory, herb layer (for the West Virginia study), and microbial communities (Zar, 2009).

### Findings

#### West Virginia study

**Figure 1:**
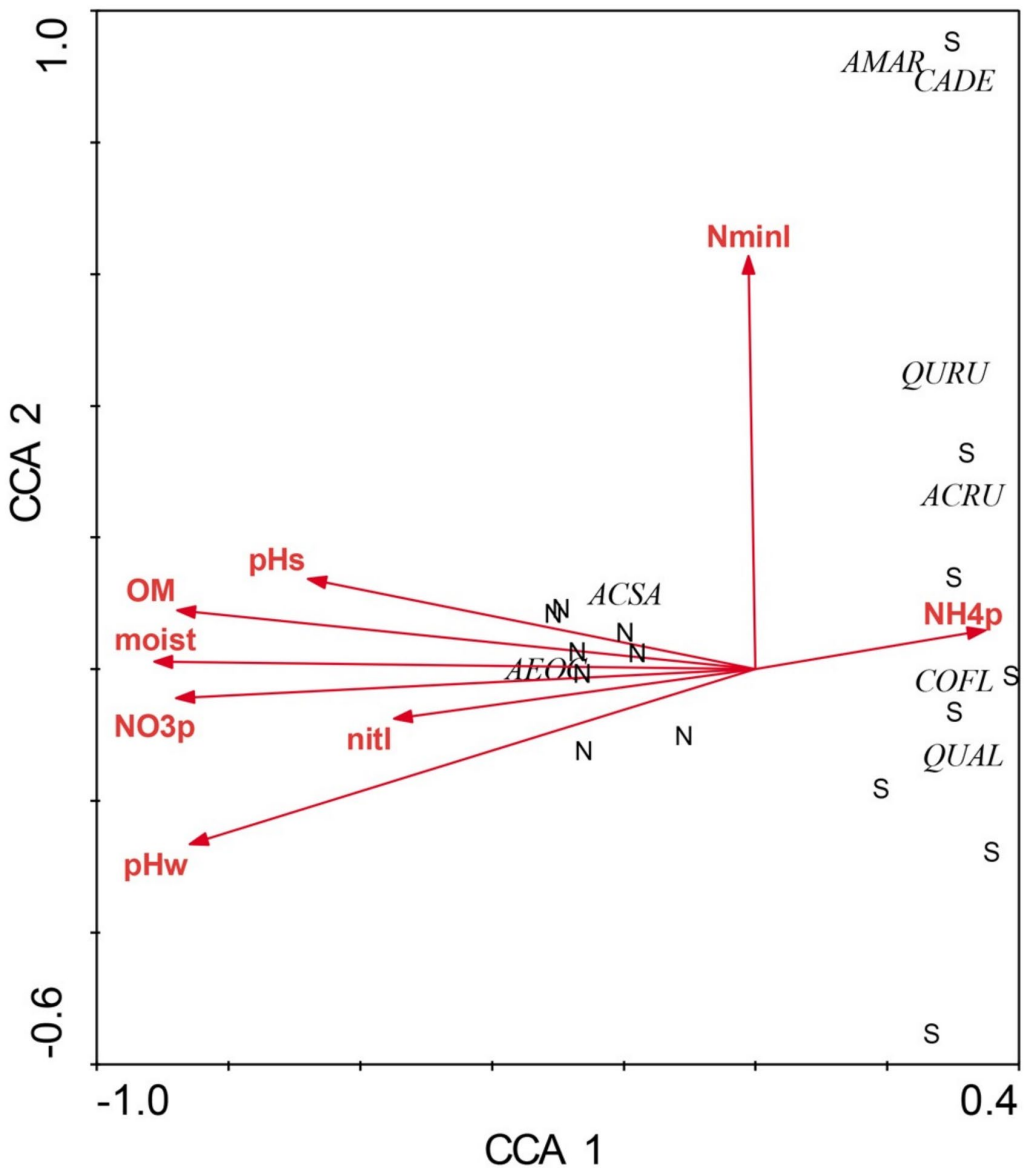
Canonical correspondence analysis of overstory species at Beech Fork Lake State Wildlife Area, WV. Plot locations in ordination space are indicated by N (northeast slopes) and S (southwest). Environmental vectors are as follows: moisture (moist), organic matter (OM), water-extractable pH (pHw), KCl-extractable pH (pHs), extractable NH4 þ and NO3_ (NH4þ and NO3, respectively), and net N mineralization and nitrification (NminI and nitI, respectively). Overstory species codes are as follows: *Aesculus octandra* (AEOC), *Acer saccharum* (ACSA), *Quercus rubra* (QURU), *Acer rubrum* (ACRU), *Cornus florida* (COFL), *Amelanchier arborea* (AMAR), *Castanea dentata* (CADE), and *Quercus alba* (QUAL). Figure taken from Gilliam et al. (2014); used by permission.

**Figure 2:**
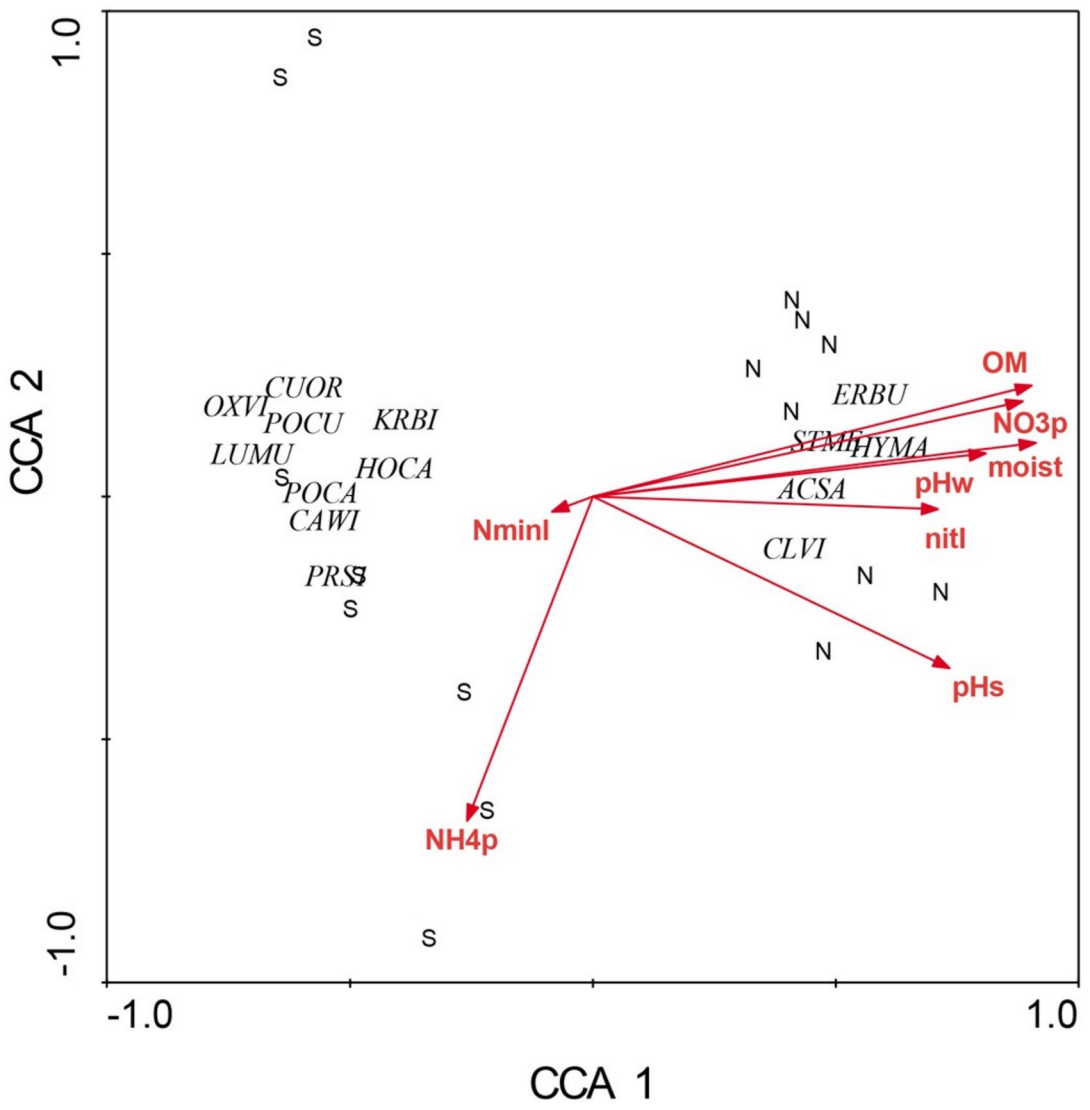
Canonical correspondence analysis of herbaceous layer species at Beech Fork Lake State Wildlife Area, WV. Plot locations in ordination space are indicated by N (northeast slopes) and S (southwest). See Figure 1 for explanation of environmental vectors. Herbaceous layer species are as follows: *Cunila origanoides* (CUOR), *Oxalis violacea* (OXVI), *Poa cuspidata* (POCU), *Krigia biflora* (KRBI), *Luzula multiflora* (LUBU), *Houstonia caerulea* (HOCA), *Potentilla canadensis* (POCA), *Carex wildenowii* (CAWI), *Prunus serotina* (PRSE), *Erigenia bulbosa* (ERBU), *Stellaria media* (STME), *Hydrophyllum macrophyllum* (HYMA), *Acer saccharum* (ACSA), and *Claytonia virginica* (CLVI). Figure taken from Gilliam et al. (2014); used by permission.

**Figure 3:**
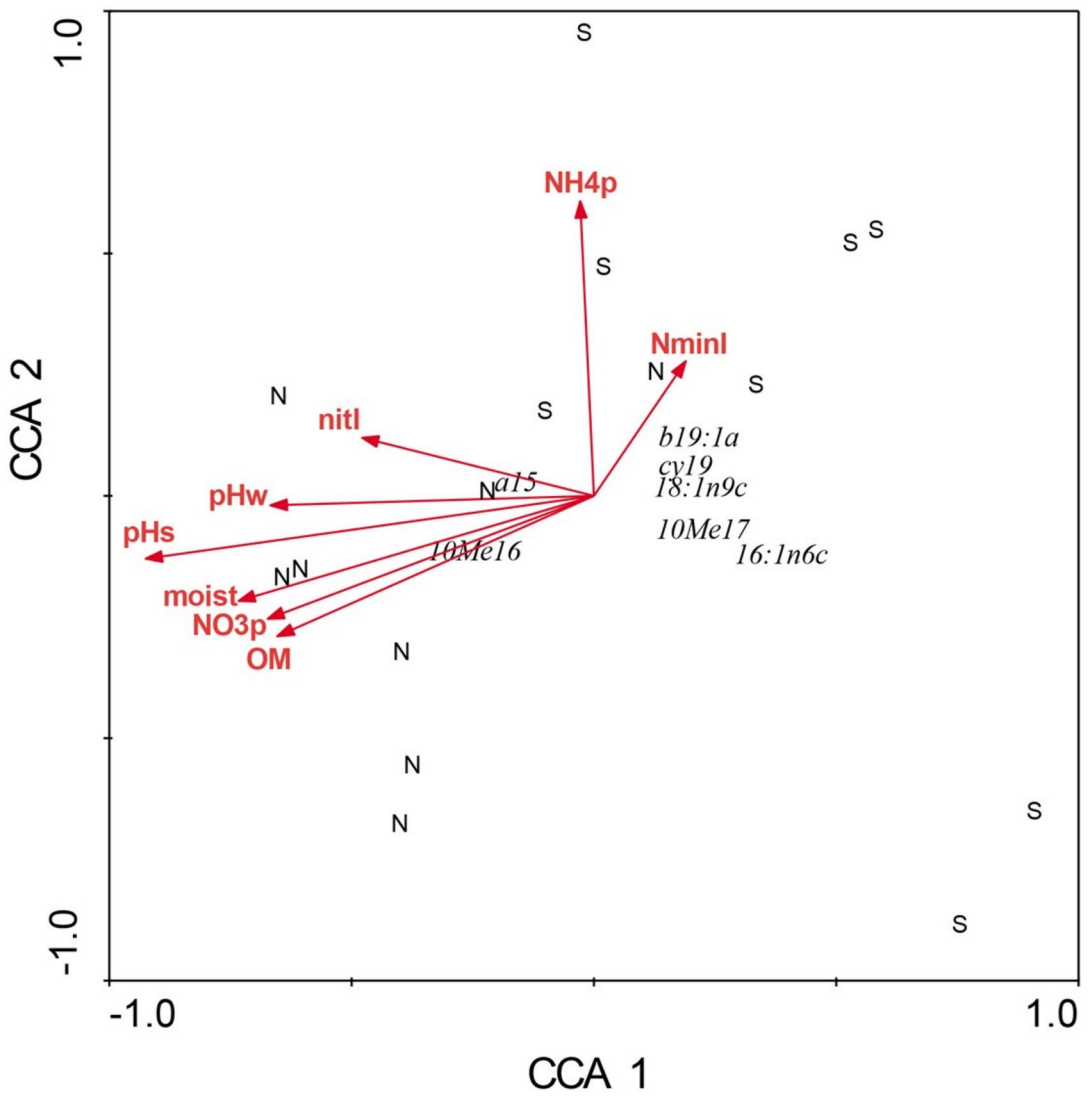
Canonical correspondence analysis of microbial composition at Beech Fork Lake State Wildlife Area, WV. Plot locations in ordination space are indicated by N (northeast slopes) and S (southwest). See Figure 1 for explanation of environmental vectors. Figure taken from Gilliam et al. (2014); used by permission.

There were profound differences in most measured variables between N-versus S-facing slopes, including soil characteristics and biotic communities. The overstory was dominated by sugar maple (*Acer saccharum*) and sweet buckeye (*Aesculus octandra*) on N slopes, whereas white oak (*Quercus alba*) dominated on S slopes (Figure 1). The herb layer of the N slope was predominantly forb species; graminoids dominated the S slope (Figure 2). Prevalent microbial groups in N-facing soils were Gram-positive and Gram-negative bacteria, whereas S-facing soils were dominated by fungal groups and Gram-negative bacteria associated with environmental stress (Figure 3). In all, the S slope exhibited numerous characteristics typical of a site with weathered, infertile soils resulting from high solar radiation (Geiger et al., 2003).

**Figure 4:**
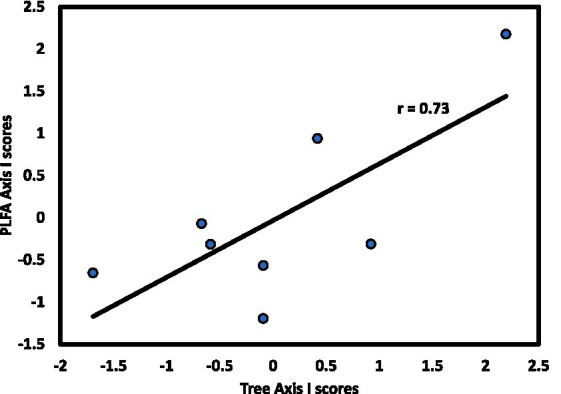
Axis I scores for CCA from the N slope of forest stands at Beech Fork Lake, WV, for tree versus microbial composition; r is the Spearman product–moment correlation coefficient for the relationship between axis scores.

**Figure 5:**
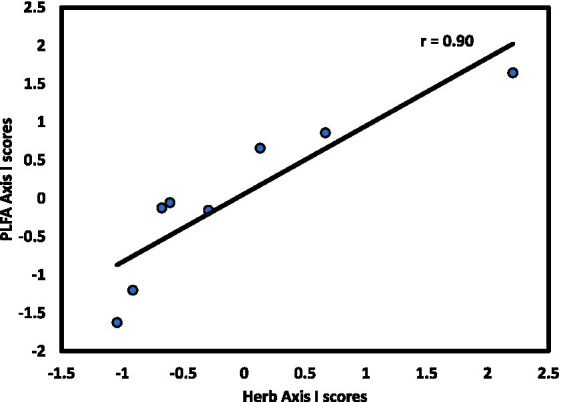
Axis I scores for CCA from the S slope of forest stands at Beech Fork Lake, WV, for herb versus microbial composition; r is the Spearman product–moment correlation coefficient for the relationship between axis scores.

The microbial community exhibited linkages with forest strata that varied by stratum and slope aspect. For the N aspect, CCA1 of the microbial community was significantly correlated with CCA1 of the overstory (*r* = 0.73, *p* < 0.05; Figure 4). For the S aspect, CCA1 of the microbial community was significantly correlated with CCA1 of the herb layer (*r* = 0.90, *p* < 0.01; Figure 5), supporting the environmental gradient hypothesis (Gilliam, 2007). On the S aspect, both the overstory and soil microbiome were influenced primarily by pH. Thoms and Gleixner (2013) also found significant relationships between tree species and soil microbial communities driven largely by variation in soil pH, working in a temperate deciduous forest in Germany, findings consistent with conclusions of an extensive review on the influence of tree species on litter and soil microbes (Prescott and Grayston, 2013).

These findings suggest that tree species on the N slope exert top-down control on microbial communities, which respond sensitively to inputs of high-quality litter from dominant tree species. Furthermore, the more weathered soils of the S slope represent bottom-up control by microbial communities; greater acidity and lower fertility select for a distinctive microbial and herb community, resulting in the observed linkage between the herb layer and soil microbiome on the S-facing slope. Burke et al. (2009) found significant effects of herb communities on soil fungi in a mature beech-maple forest. Helgason et al. (1999) concluded that forest herbs can exert a strong influence on diversity of AM fungi.

#### Florida study

**Figure 6:**
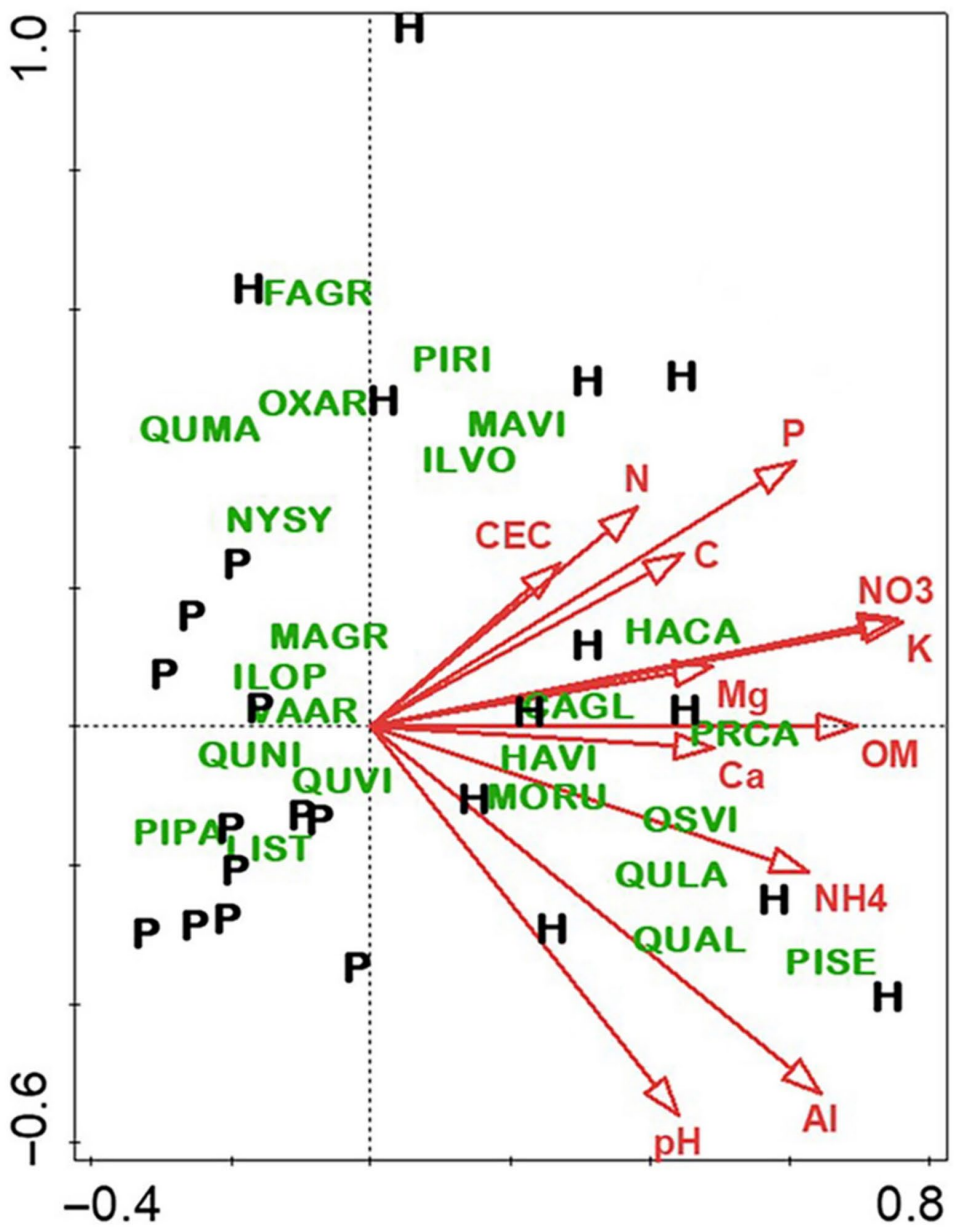
Canonical correspondence analysis of overstory species in hardwood (H) and longleaf pine-dominated stands (P). For vectors, element symbols are extractable concentrations of stated elements, “CEC” is cation exchange capacity, “OM” is organic matter, “pH” is H2O-extractable soil pH, and “C” and “N” are total C and N, respectively. Species are indicated by four-letter codes: *Carya glabra* (CAGL), *Fagus grandifolia* (FAGR), *Halesia carolina* (HACA), *Hamamelis virginiana* (HAVI), *Ilex opaca* (ILOP), *Ilex vomitoria* (ILVO), *Liquidambar styraciflua* (LIST), *Magnolia grandifolia* (MAGR), *Magnolia virginiana* (MAVI), *Morus rubra* (MORU), *Nyssa sylvatica* (NYSY), *Ostrya virginiana* (OSVI), *Oxydendrum arboreum* (OXAR), *Pinus palustris* (PIPA), *Pinus rigida* (PIRI), *Pinus serotina* (PISE), *Prunus caroliniana* (PRSE), *Quercus alba* (QUAL), *Quercus falcata* (QUFA), *Quercus laurifolia* (QULA), *Quercus marilandica* (QUMA), *Quercus nigra* (QUNI), *Quercus virginiana* (QUVI), and *Vaccinium arboretum* (VAAR). Figure taken from Gilliam et al. (2023); used by permission.

**Figure 7:**
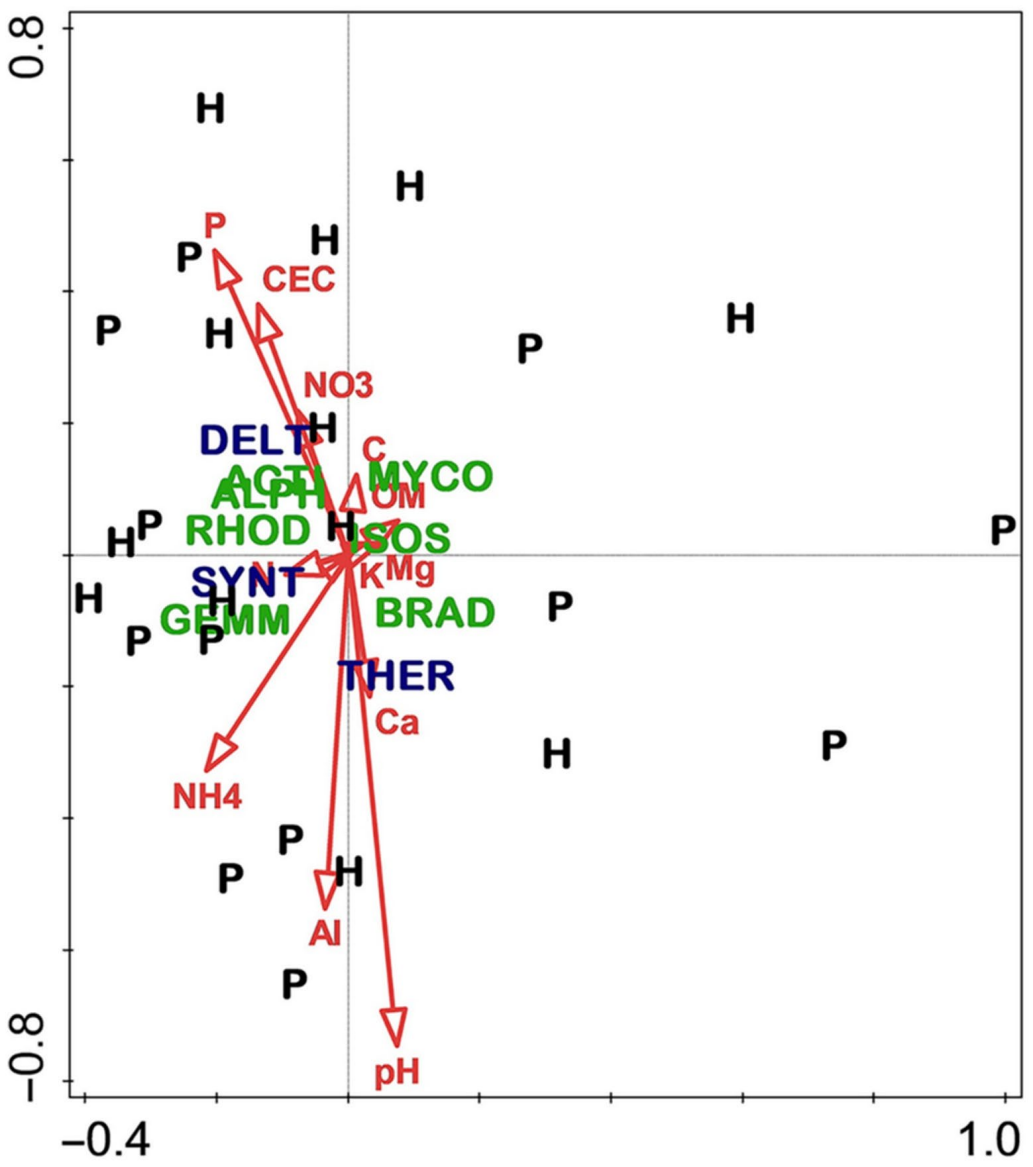
Canonical correspondence analysis of soil microbiome in hardwood (H) and longleaf pine-dominated stands (P). For vectors, element symbols are extractable concentrations of stated elements, “CEC” is cation exchange capacity, “OM” is organic matter, “pH” is H2O-extractable soil pH, and “C” and “N” are total C and N, respectively. Taxa are indicated by four-letter codes: Alphaproteobacteria (ALPH), Rhodospirillaceae (RHOD), Isosphaeraceae (ISOS), Mycobacterium (MYCO), Bradyrhizobiaceae (BRAD), Actinomycetales (ACTI), Gemmataceae (GEMM), Deltaproteobacteria (DELT), Thermogemmatisporaceae (THER), and Syntrophobacteraceae (SYNT). Violet font indicates taxa significantly different between stand types. Figure taken from Gilliam et al. (2023); used by permission.

**Figure 8:**
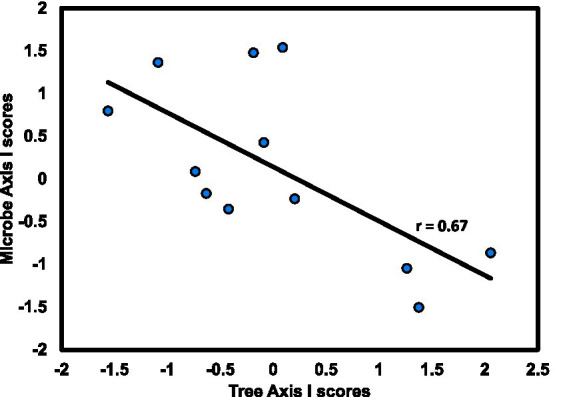
Axis I scores for CCA from hardwood stands at the University of West Florida Nature Trails for tree versus microbial composition; r is the Spearman product–moment correlation coefficient for the relationship between axis scores.

As with the slope aspect study, the differences between stand types—hardwood-versus longleaf pine-dominated—were profound for most measured variables, especially overstory composition and soil fertility. Hardwood stands were predominantly flowering magnolia (*Magnolia grandiflora*) and southern evergreen oaks, whereas pine stands were dominated by longleaf pine and live oak (*Quercus virginiana*). Although soils of both stand types were highly acidic, the hardwood soils were notably higher in fertility, especially for total and available N (Figure 6). By contrast, there were few differences in the microbiome related to stand type (Figure 7), which was surprising given the sharp differences in overstory composition and soil fertility (Figure 6). Results of the CCA involving soil microbiome revealed a strong pH gradient along the second axis (Figure 8).

Consistent with the approach of the slope aspect study, variation of linkage with stand type in this study was assessed by running CCA for each stand type separately. Although these results demonstrate overstory/microbial linkage, they also suggest that linkage at this site is stand-specific, being evident only in hardwood-dominated stands, which supports initial expectations based on earlier work in these stands (Gilliam et al., 2023). That is, linkage in the hardwood stand appears to have arisen from responses to a gradient in mineral soil pH. This is consistent with findings of Tedersoo et al. (2020), who found strong effects of soil pH and soil microbial diversity in a regional-scale analysis of Northern Europe.

## Synthesis and conclusions

Despite that these two studies addressed contrasting research questions (slope aspect versus stand type) carried out in broadly different forest types, they both sought to determine whether forest vegetation can exhibit linkage with soil microbiomes. A further distinction between them was that the slope aspect study included the forest herb community, whereas the stand type study did not. Both, however, demonstrated that linkage can exist and be demonstrated quantitatively, with the slope aspect study showing pronounced aspect-related variation in these patterns. Whereas linkage was evident for the overstory and microbiome on the N slope, it was evident for the herb layer and microbiome only on the S slope. For the stand type study, overstory/microbiome linkage was evident only for the hardwood-dominated stands. These studies also show that methodology developed and used for assessment of linkage among forest vegetation strata can be expanded to include the soil microbiome.

A principle application of these case study results to the previous review of mechanisms connecting forest vegetation and soil microbiomes would be the distinction between top-down and bottom-up interactions. For the West Virginia study, this was a clear distinction for overstory linkage in the N-facing stand (top down) versus herb layer linkage in the S-facing stand (bottom up). Top-down control is consistent with the phyllosphere microbiome as a major driver (Duan et al., 2024; Köhler et al., 2025), especially regarding soil N dynamics (Guerrieri et al., 2019, 2024). By contrast, bottom up control comprises belowground processes, including mycorrhizae (Zubek et al., 2024), rhizosphere microbial activity (Han et al., 2022), and even soil weathering (Lopez and Bacilio, 2020). The latter forms an important connection between the two case studies, as the Florida study displayed linkage driven by soil pH, which both decreases with weathering and sensitively affects soil bacterial communities (Guo et al., 2023), clearly another example of bottom up control.

As demonstrated herein, considerable current work has focused not only on the soil microbiome of forest soils, but also on the close, often causal, relationship between soil microbes and forest vegetation. This is reassuring from a scientific point of view, especially considering the sensitive nature of soil microbiomes to climate change, and more specifically to changes in the frequency or intensity of extreme climatic events (Knight et al., 2024). Climate change-related shifts in forest composition have long been predicted (Gilliam, 2016).

Climate change, however, is not the only threat to these close connections between forest vegetation and soil microbiomes. Some threats comprise disturbances of both anthropogenic and natural origin, such as logging and fire (Bowd et al., 2020), as well as biotic pressures, including diseases (Baldrian et al., 2023), herbivory (Segar et al., 2022), and invasive species (Burke et al., 2019; Duchesneau et al., 2021).

Although it has not been the focus of this review to synthesize what is known regarding the effects of disturbance on vegetation/soil microbiome interactions, such effects comprise an important facet in our understanding of the essential role of the soil microbiome in forest ecosystems. Among the more profound effects of disturbance is its influence on spatial heterogeneity, a response which itself is potentially quite complex. For example, some disturbances, such as excess N deposition, can decrease spatial heterogeneity (Gilliam et al., 2011), others, such as fire and harvesting, can increase spatial heterogeneity (Holden and Treseder, 2013). Whichever the outcome, both have potentially profound effects on the soil microbiome and its interaction with forest vegetation.

Moving forward, future research must also consider how both forest plant species and soil microbes will respond to ongoing changes in all of these threats, most of which are intimately interconnected. Such work will be essential in informing forest policy and management decisions. Consideration of linkages between forest strata and soil microbiomes should be part of that research.
